# Is nonangiogenesis a novel pathway for cancer progression? A study using 3-dimensional tumour reconstructions

**DOI:** 10.1038/sj.bjc.6603039

**Published:** 2006-04-18

**Authors:** O Adighibe, K Micklem, L Campo, M Ferguson, A Harris, R Pozos, K Gatter, F Pezzella

**Affiliations:** 1 Laboratory of Neurogenetics, National Institute on Aging, National Institute of Health, 35 Convent Drive, Bethesda, MD 20892, USA; 2 Cancer Research UK Tumour Pathology Group, Nuffield Department of Clinical Laboratory Science, University of Oxford, John Radcliffe Hospital, Headington OX3 9DU, Oxford, UK; 3 Cancer Research UK Medical Oncology Unit, Churchill Hospital, Old Road, OX3 7LJ, Oxford, UK; 4 San Diego State University, 5250 Campanile Drive, San Diego, CA 92182-1931, USA; 5 Minority International Research Training Program, Division of International Training and Research, Fogarty International Center, National Institutes of Health, Building 31, Room B2C39, 31 Center Drive, MSC 2220, Bethesda, MD 20892-2220, USA

**Keywords:** lung cancer, angiogenesis, 3D reconstruction

## Abstract

The nonangiogenic lung tumour is characterized by neoplastic cells co-opting the pre-existent vasculature and filling the alveoli space. 3-Dimensional reconstruction of the tumour reveals that this particular tumour progresses without neovascularization and there is no major destruction of the lung's architectural integrity.

In recent years, the role of angiogenesis in neoplastic growth has become controversial. Initially, it was thought that the formation of new capillaries (neovascularization) usually mediated by angiogenic molecules released by tumour cells and activated macrophages was essential for all tumour growth (Perez-Atayde *et al*, 1997; Passalidou *et al*, 2002; Pezzella *et al*, 1997). Now there is growing evidence that in certain situations tumours can obtain sufficient blood supplies from pre-existing vascular beds to grow without angiogenesis. This form of neoplastic growth has been termed nonangiogenesis (Holash *et al*, 1999; Pezzella *et al*, 2001). A pattern of nonangiogenic growth has been described by Wesseling *et al* (1994) Al in glioblastoma multiforme and by our group in a large series of non-small cell lung carcinoma (Pezzella *et al*, 2001). In the latter, neoplastic cells filled the alveolar spaces (Pezzella *et al*, 2001) and showed no evidence of vascularization but grew by co-opting pre-existing pulmonary blood vessels. These nonangiogenic cases made up about 16% of the series (Pezzella *et al*, 1997) and were more aggressive clinically than the predominant angiogenic tumours (Pezzella *et al*, 2001).

To date these studies have given little consideration to the fact that tumour growth both angiogenic and nonangiogenic occurs in 3 dimensions. In this study, we have used computer aided three-dimensional (3D) reconstructions to demonstrate the distinct differences in vascularity and morphology between the nonangiogenic and angiogenic lung tumours. We also show that in nonangiogenic tumours the integrity of the lung architecture is retained while these alveolar entrapped neoplastic cells continue to thrive without producing new vessels of their own.

## MATERIALS AND METHODS

Our model for this reconstruction is non-small cell carcinoma of the lung. Both angiogenic and nonangiogenic tissues are embedded in paraffin. The region of the tissue used for the study is 2 cm in depth. In all, 200 5 *μ*m thick sections of paraffin-embedded tissue were cut from each case and mounted on slides coated with poly-L-lysine.

### Clinical details of tissue samples

Primary non-small cell lung cancer tissues (angiogenic and nonangiogenic) were obtained with informed consent from two patients who underwent radical surgical resection. The normal tissue was obtained from a patient with a lung secondary who also underwent radical resection. This research project was approved by the local ethical committee. The diagnosis was established on routine formalin-fixed paraffin-embedded material. One section was stained with haematoxylin and eosin to verify the presence of viable tumour; another was immunostained for CD34 to assess the vascular pattern as previously described.

### Antigen retrieval

Antigen retrieval is as previously described by Pileri *et al* (1997).

### Immunocytochemistry

Blood vessels and cytoskeleton on the dewaxed sections were identified by simultaneous immunostaining with 1 : 500 dilution of anti CD34 antibody QBEnd/10 (DAKO, UK) and 1 : 25 dilution of a pan-cytokeratin rabbit polyclonal antiserum (Novacastra, UK). The primary antibody staining was allowed to incubate for 1 h, followed by a 5 min wash with TBS. Immunodetection was carried out for blood vessels and cytoskeleton by another simultaneous 1 h incubation with 1 : 200 dilution of both Alexa Fluor 488 goat anti-rabbit and Alexa Fluor 568 goat anti-mouse (Molecular Probes, USA). The immunostained sections were washed in TBS for 5 min and then mounted in antifade Dako Fluorescent mounting medium (DAKO, USA) containing a 1 : 500 dilution of 4′,6-diamidine-2′-phenylindole dihydrochloride DAPI (Roche Molecular Biochemicals, UK).

### Image acquisition and processing

Specific areas on immunostained sections were examined with the × 10 Plan fluor objective lens of a Nikon Eclipse E600 Fluorescence microscope and photographed by an interfaced Zeiss Axiocam (Germany). Photographed images were then captured by Axiovision software in an interfaced computer. The emitting fluorescence signals were selected, respectively, by a group of filters, Dappi, FITC, Tx Red and exhibited with a resolution of 1300 by 1030 pixels of Red Green Blue (RGB). Acquired images were then batch converted from Axiovision to Photoshop where all three colours of individual images were superimposed and consecutive slide images were then stacked on top of each other for 3D reconstruction/restoration of the spatial orientation of section images. The stacked image was then imported to Imaris (Bitplane) software for the 3D rendering of the images.

## RESULTS

As we expected, it was evident from the 2D picture and 3D rendering (supplement) that the architectural contour of the nonangiogenic lung tumour is a replica of the normal lung. When we compared the vascular network staining (CD34) of the normal to the nonangiogenic lung, they appeared to be indistinguishable from each other. The only difference between the normal and the nonangiogenic lung is seen when the cytokeratin staining is superimposed on CD34 staining; then the spongy like morphology of the normal lung is tumour filled in the nonangiogenic lung marked by the green cytokeratin fluorescence colour delineating these tumour cells. These nonangiogenic tumour cells are growing within the boundaries or confines of the already existing blood vessels of the lung and this pattern of the tumour growth very obviously extends beyond the surface to deep within the tissue as depicted by the 3D (supplement). Also, it is quite clear from the 3D picture (supplement) that this tumour growth pattern seen in the nonangiogenic is very distinct from the angiogenic form. The angiogenic cancer has no defined pattern of growth. Its vascular network is chaotically distributed with many blood vessels dilating twice to triple the size of adjoining vasculature. Also some of these angiogenic blood vessels appear to have blind endings while the eruption of others incite destruction of pericytes leading to the leakiness of blood vessels usually observed in angiogenic tumours.

## DISCUSSION

**Figure 1:**
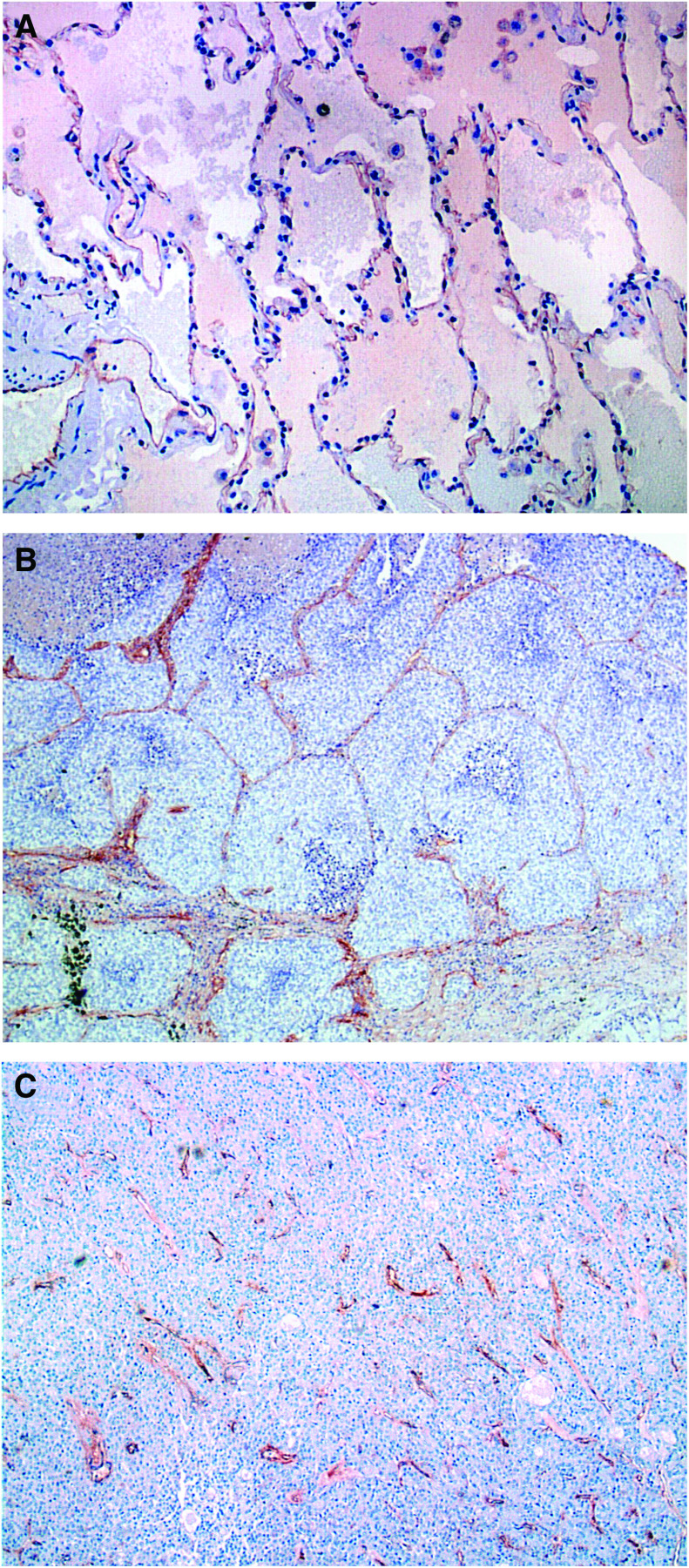
(**A**) H&E slide of normal lung with a spongy appearance of the alveoli membrane, which are characteristically lined by thin blood vessels. (**B**) Nonangiogenic lung tumour on H&E with the filling of alveoli by neoplastic cells, a lack of parenchymal destruction, as well as an absences of neovascularization and tumour associated stroma. The only blood vessels present are those of the alveoli septa. (**C**) H&E of angiogenic lung tumour with the hallmark destruction of normal lung architecture with the production of tumour-associated stroma and of new blood vessels erratically scattered within a sea of neoplastic cells.

For this experiment, we chose to use the fluorescence staining technique because its advantage over regular optical microscopy is the convenience of selectively observing the structure of the nuclei (blue), vasculature (red) and cytokeratin (green) staining individually. With this technique, there is also the option to superimpose any of the image staining upon each other. This way, we were able to trace specific regions and easily construct the 2D (Figure 1) and 3D morphology of the normal, nonangiogenic and angiogenic lung tumours.

What we have done with this study is to provide morphological evidence that a nonangiogenic tumour phenotype does exist. This then raises the question of whether this nonangiogenic phenotype has relevant biological differences to the angiogenic form. If this were the case, it would have major implications with respect to any antiangiogenic treatments.

**Figure 2:**
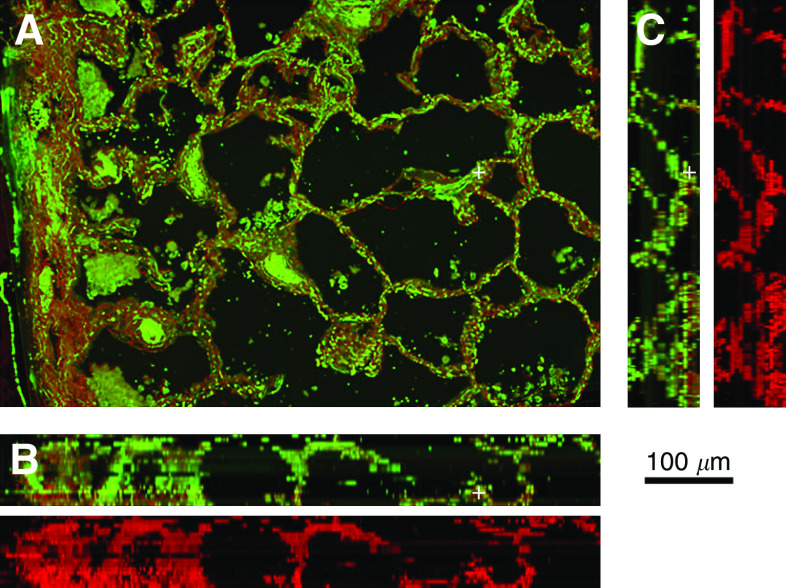
(**A**) 2D rendering of a normal lung. (**B** and **C**) Orthogonal views of the normal lung with the staining of the vascular network followed by the superimposition of cytokeratin staining.

**Figure 3:**
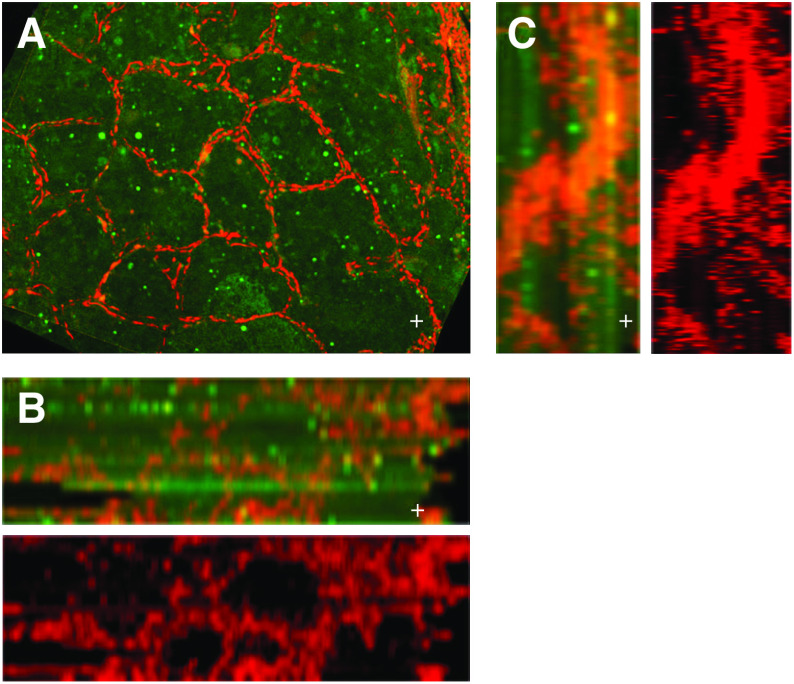
(**A**) 2D of Nonangiogenic cancerous lung with blood vessel lining still intact and similar to that of the normal lung except that in the former, the alveoli are filled with tumours. (**B**) 2D orthogonal view showing that the blood vessel staining of the nonangiogenic lung looks like a replica of a normal lung with no destruction to the vascular network. Followed by the superimposition of the cytokeratin staining showing neoplastic cell filled alveoli. (**C**) 2D orthogonal view of the nonangiogenic tumour of a large long blood vessel that remains intact and snakes through from the surface deep into the tissue with not evidence of vascular eruption.

**Figure 4:**
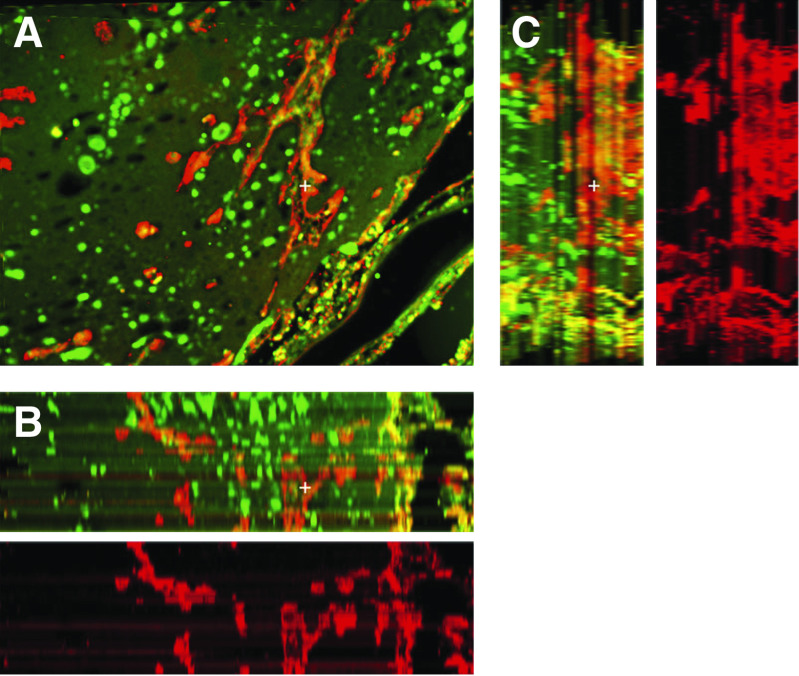
2D of angiogenic lung showing tortuous blood vessels of heterogeneous sizes. There evidently is no retention of the original architecture of the normal lung's blood vessel as in the nonangiogenic tumour.

Studies in mice have shown that not all experimental metastases respond to antiangiogenic agents (Breast Cancer Progression Working Party, 2000). Clinical trials results suggest the possibility that some of these unresponsive tumours are of a distinct phenotype from the responsive angiogenic tumours (Gasparini *et al*, 2005). Studies in our laboratory have shown evidence of a variant lung tumour phenotype (the nonangiogenic form) that grows filling the alveoli without the neovascularization of angiogenesis. The idea that these alveolar filling neoplastic cells are mostly likely thriving through co-opting of the pre-existent normal lung blood vessels is supported by our 2D (Figure 2) and 3D models (Figure 3) (supplement). In contrast, the angiogenic tumours have no distinct patterns of growth and consist of heterogeneous amalgams of blind ended, tortuous blood vessels of varied sizes (Figure 4).

Having established the existence of a putative nonangiogenic tumour phenotype, we propose that a possible explanation for this phenomena could be as a result of the evolution of tumour genes via clonal selection. Owing to hypoxic events that frequently occur during cancer cell progression, genes such as those governing efficient regulation of oxygen homeostasis could be properties clonally coselected for by evolving cancer cells (Pugh and Ratcliffe, 2003). This suggestion is supported by Hu *et al* (2005) who have described finding higher levels of genes coding for proteins involved in mitochondrial metabolism in nonangiogenic tumours. It is also known that clonal chromosomal changes found in malignant tumours have a strong correlation with tumour morphology (Gisselsson *et al*, 2001; Gisselsson, 2002). Hence, we suggest that by means of natural selection either through interaction between the microenvironment and the variability inherent in cell populations (Kozlov, 1996) tumours cells have evolved to this nonangiogenic form in an effort to establish a survival advantage by being able to highly regulate their mitochondria and co-opting pre-existing vessels.
